# Inductive Reasoning Differs Between Taxonomic and Thematic Contexts: Electrophysiological Evidence

**DOI:** 10.3389/fpsyg.2019.01702

**Published:** 2019-07-25

**Authors:** Fangfang Liu, Jiahui Han, Lingcong Zhang, Fuhong Li

**Affiliations:** ^1^School of Psychology, Jiangxi Normal University, Nanchang, China; ^2^School of Educational Science, Minnan Normal University, Zhangzhou, China

**Keywords:** inductive reasoning, distance effect, thematic contexts, taxonomic contexts, event-related potential (ERP)

## Abstract

Inductive reasoning can be performed in different contexts, but it is unclear whether the neural mechanism of reasoning performed in a thematic context (e.g., bee has x, so honey has x) is the same as that performed in a taxonomic context (e.g., bee has x, so butterfly has x). In the present study, participants were required to judge whether a conclusion was acceptable or not based on its premise, for which the taxonomic or thematic distances between premise and conclusion objects were either far or near. The Event related potential (ERP) results indicated that the effect of context (taxonomic vs. thematic) was initially observed in the P2 component; while the distance effect (far vs. near) was observed in N400 and late components. Moreover, the distance effect on thematic-based inductive reasoning was found in the anterior regions, while the distance effect on taxonomic-based inductive reasoning conditions was found in the posterior regions. These results support the view that inductive reasoning is performed differently under different semantic contexts.

## Introduction

Inductive reasoning is a complex, high-level cognitive process that is pervasive in human life. It can generate new knowledge based on limited information and extend to new situations. This ability can effectively improve human learning (Babcock and Vallesi, 2015). For example, if we know that bears have sesamoid bones, we might infer that moose are more likely to have sesamoid bones than salmon because moose are more biologically similar to bears (Kemp and Tenenbaum, 2009). In recent years, inductive reasoning and its cognitive neural mechanism have widely been studied using various tasks, with a number of model (Neely, 1991; Goel and Dolan, 2004; Li et al., 2009b, 2013; Liang et al., 2010, 2014; Paulsen et al., 2010; Bruffaerts et al., 2013; Bonnefond et al., 2014; Khatoonabadi et al., 2016). The two most popular accounts of inductive reasoning are the similarity-based model (Osherson et al., 1990; Sloutsky and Fisher, 2004) and concept-based model (Gelman and Davidson, 2013).

The similarity-based model suggests that the inferences are driven by perceptual similarity. For example, if items A and B have similarities, then people may make the following reasoning “If item A has property X, then item B also has property X” (Sloutsky et al., 2007). Notably, the precondition to perform the above reasoning process between items A and B is the similarity between the items or shared membership in a category. That is, there is a certain relationship between A and B in a semantic context, which is based on the similarity of the in taxonomic relation. The taxonomic relation refers to an overlap in the features or meaning of words, which includes items of the same superordinate category (e.g., mammal, with members such as panda, antelope, dog, cat, cow, etc., Sachs et al., 2008a,b). Various studies on inductive reasoning have supported the role of taxonomic relation in the reasoning process (Rips, 1975; Carey, 1985; Gelman et al., 1986; Gelman and Markman, 1987; Keil, 1987; Osherson et al., 1990; Sloman, 1993; Smith and Jones, 1993; Medin et al., 1997; Lin and Murphy, 2001; Xiao, 2009; Liang et al., 2010; Babcock and Vallesi, 2015; Long et al., 2015).

In contrast, the concept-based model proposes that inductive reasoning is based on existing knowledge or concepts. In other words, people tend to make inductive reasoning based on common conceptual property that is often denoted by linguistic labels, while ignoring perceptual similarities between items (Gelman, 2003). As one of the conceptual properties, thematic relation is based on externally or complementary related items within scenarios or events (e.g., bee–honey), which shares an associative relationship but not perceptual features (Lin and Murphy, 2001). Recently, a number of studies have begun to reveal the role of thematic relation in categorization and inductive reasoning (Lin and Murphy, 2001; Kalénine et al., 2009; Schwartz et al., 2011; Lewis et al., 2015).

Some researchers have investigated how the thematic relations are processed in inductive reasoning and compared this to the processing of taxonomic relations (Lin and Murphy, 2001; Xiao, 2009). For example, Lin and Murphy (2001) explored whether thematic relations would promote inductive reasoning in the presence of taxonomic relations. They found that if thematic relations can be coherent and meaningful (Bacteria, for example, have properties that rely more on external contacts with items that co-occur in space and time than on internal taxonomic relations), then subjects might be willing to use them as the basis for inductive reasoning, for cases in which people make inductive reasoning primarily based on thematic rather than taxonomic relations. Shafto et al. (2007) examined the effects of the time pressure on inductive reasoning under different contexts (taxonomic vs. ecological vs. unrelated). They found that the performance of taxonomic inferences is significantly better than that of ecological inferences. Moreover, Shafto et al. (2007) showed that the knowledge of taxonomic relation is more easily acquired in the reasoning process than that of thematic relation. Other studies have found that the processing of thematic relation requires fewer cognitive resources than the processing of taxonomic relation in reasoning tasks (Sachs et al., 2008a; Kalénine et al., 2009; Lewis et al., 2015). Recently, some researchers tried to elucidate the neural basis for these differences. For example Kalénine and Buxbaum (2016), found that taxonomic processing activates the bilateral visual areas, and thematic processing recruited a bilateral temporo-parietal network including the inferior parietal lobules (IPL) and middle temporal gyri (MTG). Accordingly, they suggested that taxonomic relations rely on perceptual processes while thematic relations rely on event/action processing (Kalénine and Buxbaum, 2016).

Existing studies have primarily adopted imaging methods to explore the neural difference between taxonomic- and thematic-based inductive reasoning (Krueger and Clement, 1996; Kalénine et al., 2009; Xiao, 2009; Kalénine and Buxbaum, 2016). However, the temporal dynamics of brain activation associated with the difference of thematic and taxonomic relations on inductive reasoning remains unaddressed. Thus, the purpose of the present study was to investigate the electrophysiological distinction between these two types of inductive reasoning. Particularly, we tested whether distance effects on the processing of taxonomic- and thematic-based semantic relations in inductive reasoning were differently reflected in brain. We designed four conditions, including taxonomic-far, taxonomic-near, thematic-far, and thematic-near conditions. In each trial, a premise and a conclusion were sequentially presented. Participants had to decide whether the conclusions were acceptable or not.

First, the high temporal resolution of ERP enables us to examine the timing of the brain's processing of context and distance during an inductive reasoning task. We presumed that the recognition of the relation type between items denoted by words is more likely to be reflected in the access and representation of categorical information corresponding to a word, which is the premise of relationship judgments. Accordingly, the effect of relation type may be observed in earlier time windows such as the P2 component, which is not only related to lower-order perceptual decoding, but also to higher-order semantic processing (Liang et al., 2010; Paulsen et al., 2010; Bonnefond et al., 2014; Chen et al., 2015b). On the contrary, distance effects may be observed in the integration of linguistic relations between the premise and conclusion, mainly in semantic processing. Accordingly, distance effects may be observed in the later time window such as the N400, which is related to semantic integration (Long et al., 2015; Wamain et al., 2015).

Second, measuring electrophysiological responses (such as amplitude and latency) to context also help us to investigate whether different types of inductive reasoning require different amounts of cognitive resources at different time windows. Previous studies have found that the cognitive demand during taxonomic and thematic judgments may differ depending on whether the object is an artifact such as a hammer or a natural object such as a cherry (Kalénine et al., 2009, 2012; Lewis et al., 2015; Kalénine and Buxbaum, 2016; Vivas et al., 2016). When the object is an artifact, the thematic relations are processed more easily than the taxonomic relations (Kalénine et al., 2009, 2012; Lewis et al., 2015; Kalénine and Buxbaum, 2016). To further support this view, we used artifacts as objects in the present study and expected better performance in thematic judgment as compared to taxonomic judgment. Moreover, we predicted that taxonomic judgment might evoke larger amplitude than thematic judgment in the P2 time window, while the thematic judgment might evoke larger amplitude than taxonomic judgment in the N400 time window. This prediction is based on evidence that taxonomic relations rely on perceptual processes while thematic relations rely on event/action processing (Kalénine et al., 2009).

Third, in the present study, the distance of taxonomic and thematic relationships is also closely related to semantic integration and processing. Therefore, we predicted that N400 will be observed in the inductive reasoning under these two contexts since far distance requires more cognitive resources than near distance in the process of semantic integration (Lewis et al., 2015). Therefore, we hypothesized that for both thematic and taxonomic contexts, the far distances may evoke a greater N400 than near distances.

## Methods

### Participants

Sixty-four healthy undergraduate students (aged 18–25 years) rated the experimental materials in a pilot test. Another 29 healthy undergraduate students (19 female, mean age = 20.03 years, range: 18–24 years, *SD* = 1.45) participated in the formal experiment, with five excluded due to excessive eye movements. Thus, 24 participants remained in the ERPs. All participants were right-handed with normal or corrected-to-normal vision. All participants provided written informed consent and were monetarily compensated. The experiment was approved by the ethics review board of Jiangxi Normal University.

### Stimuli and Experimental Procedure

In the pilot experiment, participants were required to rate the strength of thematic and taxonomic relations for each word pair on a 7-point scale (7: very relevant, 4: moderate relevance, 0: completely irrelevant). In the present study, each condition had 128 trials that appeared with same frequency. Four types of conditions appeared randomly to avoid the expectation of subjects. According to previous research, we translated the word pairs into Chinese and selected the artificial words (Lau et al., 2008; Kalénine et al., 2009; Maguire et al., 2010; Bonnefond et al., 2014; Sloutsky et al., 2015). See S-Table 1 Appendix for all word pairs.

Four types of word-pairs were designed, with a total of 256 pairs of words. Sixty-four pairs had a thematic-far relation, whereby the two words were thematically related with a far distance (e.g., saw vs. rebar); 64 pairs had a thematic-near relation, whereby the two words were thematically related with a near distance (e.g., saw vs. wood); 64 pairs had a taxonomic-far relation, whereby the two words were taxonomically related with a far distance (e.g., saw vs. pliers); and 64 pairs had taxonomic-near relation, whereby the two words were taxonomically related with a near distance (e.g., saw vs. axe).

Finally, the 64 pairs of words in the four conditions were retained and used in the formal experiment. A paired-*t* test indicated that the thematic-near word pairs (*M* = 6.08, *SD* = 0.28) were significantly different from the thematic-far word pairs (*M* = 4.39, *SD* = 1.05) in terms of thematic relation, *t*_(1, 126)_ = 12.48, *p* < 0.01. The taxonomic-near word pairs (*M* = 5.62, *SD* = 0.46) differed significantly from the taxonomic-far word pairs (*M* = 3.77, *SD* = 0.86) in terms of taxonomic relation, *t*_(1, 126)_ = 16.78, *p* < 0.01.

The formal experiment was a category-based induction task (Long et al., 2015). In the current study, the four conditions have the same premise while these conditions have different four conclusion items (see Table 1 for an example). To control the effect of extra- variables, for both taxonomic and thematic conditions, we utilized the artifact premises rather than the animal or natural premises in four conditions. The following are the examples. At the end of each trial, a question mark indicated that participants should decide whether the conclusion was acceptable or not based on the premise. The premise was a sentence that stated that an object had a novel property (e.g., X1). The novel property was presented by mixing a capital letter with an Arabic number ranging from 1 to 9 (e.g. X1), which served as the blank property. The blank property was also regarded as a meaningless property, which would reduce the influence of background knowledge on reasoning.

**Table 1:** Example of arguments used in different conditions.

**Conditions**	**Premise**	**Conclusion**
Taxonomic-near	Saw has X1	Ax has X1?
Taxonomic-far	Saw has X1	Pliers has X1?
Thematic-near	Saw has X1	Wood has X1?
Thematic-far	Saw has X1	Rebar has X1?

**Figure 1:**
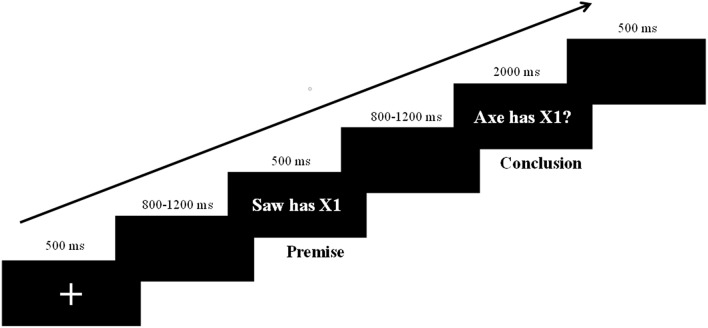
Experimental design and the procedure for a trial.

Four types of arguments (Taxonomic-Near, Taxonomic-Far, Thematic-Near, Thematic-Far) were presented sequentially on a computer screen and each argument was repeated twice. The entire formal experiment comprised 512 trials (128 in each condition). As shown in Figure 1, a fixation cross was presented in the center of a black screen for 500 ms at the beginning of each trial. After a blank screen (800–1,200 ms), the premise was presented for 500 ms, followed by another blank screen for 800–1,200 ms. Next, the conclusion appeared on the screen and remained until participants responded. Participants were instructed to respond to the conclusions as rapidly and accurately as possible. They were asked to press the “F” key for “yes” and the “J” key for “no” using the left or right index finger, respectively. The keys for different responses were counterbalanced across participants. Twenty-five practice trials were completed before the test to familiarize participants to the procedure. The arguments used in practice trials were not included in the formal experiment (see Figure 1 for experimental procedure).

### ERP Recordings and Statistical Analyses

Electrophysiological activity was recorded using a 64-channel EEG system (Brain Products GmbH, Munich, Germany), with the reference electrodes on the left and right mastoids. An electrode placed under the right eye (for electrooculography; EOG) allowed the monitoring of blinks and vertical eye movements. The impedance of all electrodes was maintained below 5 kΩ. Raw data were band-pass filtered between 0.01 and 100 Hz and digitized at a sampling rate of 500 Hz. Trials with EOG artifacts (a mean EOG voltage exceeding ±80 μV), and those contaminated with artifacts due to amplifier clipping, bursts of electromyographic activity, or peak-to-peak deflections exceeding ±80 μV were excluded from averaging.

Data were collected continuously and analyzed off-line using Brain Vision Analyzer Software 2.1 (BrainProducts, Munich, Germany). Frequencies lower than 0.01 Hz or higher than 30 Hz were digitally filtered (the filter slope 24 dB per octave). The analysis epoch was 1,000 ms with respect to the averaged voltage over the 200-ms epoch before the onset of the conclusion stimulus. The ERP waveforms were time-locked to the onset of the conclusion stimuli. The averaged epoch for the ERPs to the conclusion stimuli, including a 200-ms pre-stimulus baseline, was 1,200 ms. According to visual inspection of the grand average waveforms and previous studies (Liang et al., 2010; Long et al., 2015), the P2 (190–240 ms) (Liang et al., 2010, 2016; Chen et al., 2015b), N400 (360–440 ms) (Long et al., 2015), and late negative component (LNC) (500–800 ms) (Liang et al., 2010, 2016; Long et al., 2015) at 15 electrode sites (F3, Fz, F4, FC3, FCz, FC4, C3, Cz, C4, CP3, CPz, CP4, P3, Pz, and P4) (Hickey et al., 2010) were analyzed. The mean amplitudes of each component were analyzed using a 2 (distance: far vs. near) ×2 (context: thematic vs. taxonomic) ×3 (laterality: left, middle, right) ×5 (frontality: frontal, frontal-central, central, parietal-central, parietal) repeated measures ANOVA. As previous studies about reasoning, we adopted the appropriate correction criterion; the *p*-values in the ANOVA were corrected with the Greenhouse-Geisser correction when necessary and multiple comparison was corrected with Bonferroni criterion (Bigman and Pratt, 2004; Bright and Feeney, 2014).

## Results

### Behavioral Results

**Figure 2:**
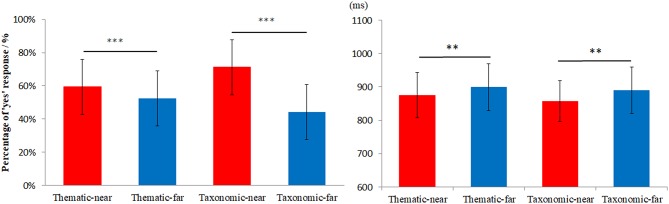
The behavioral results for different conditions. The left side is the percentage of “yes” responses and the right side is the reaction time. **p < 0.005, ***p < 0.001.

Figure 2 shows the frequency of “yes” responses (left) and RT (right). The repeated measures ANOVA showed that the frequency of “yes” responses for the near distance was higher than that of the far distance, *F*_(1, 28)_ = 6.92, *p* < 0.05, η^2^ = 0.59. There was no main effect of context, *F*_(1, 28)_ = 1.02, *p* = 0.321, η^2^ = 0.038. There was a marginally significant interaction between context and distance, *F*_(1, 28)_ = 3.16, *p* = 0.087, η^2^ = 0.108. The simple effect analysis indicated that frequency of ‘yes’ responses for thematic-far was higher than for taxonomic-far at a marginal significance level (*p* = 0.065).

For the analysis of RT, any outlier (beyond two standards of mean RT) were excluded for each subject. The percentage of trials with outlier RTs was approximately 1.62, 1.27, 1.24, and 1.37% for thematic-near, thematic-far, taxonomic-near, and taxonomic-far, respectively. The repeated measures ANOVA showed a main effect of distance [*F*_(1, 28)_ = 16.54, *p* < 0.001, η^2^ = 0.37], with faster responses in the near distance than in the far distance. Whereas, the main effect of context was not significant [*F*_(1, 28)_ = 0.37, *p* = 0.54, η^2^ = 0.013]. There was no interaction between the context and distance [*F*_(1, 28)_ = 0.16, *p* = 0.69, η^2^ = 0.006].

### ERP Results

#### P2 (190–240 ms)

**Figure 3:**
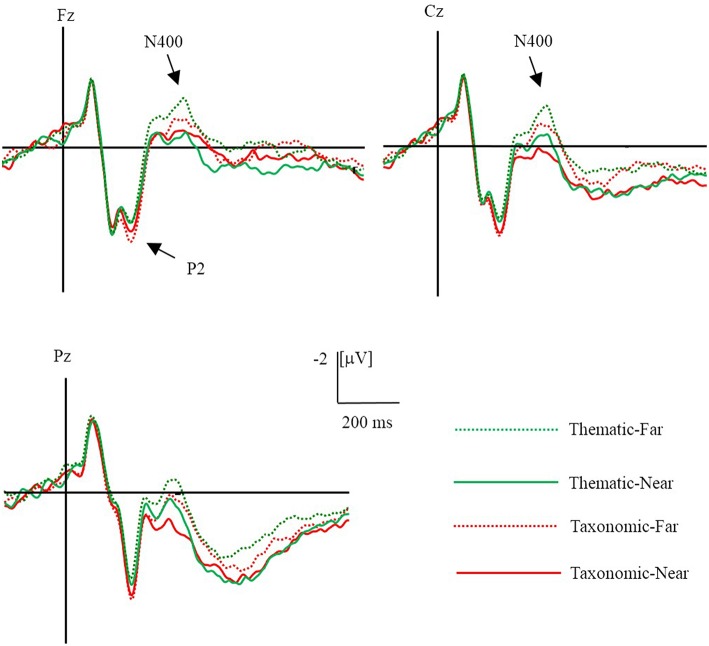
Grand averaged (*n* = 24) ERPs evoked by different conditions. The green dot line is far thematic, the green solid line is near thematic, the red dot line is far taxonomic and the red solid line is near taxonomic.

The ERPs evoked by the conclusion in the different conditions are shown in Figure 3. There was a main effect of context, *F*_(1, 23)_ = 25.96*, p* < 0.01, η^2^ = 0.53, in which taxonomic relations elicited a larger P2 amplitude than the thematic relations. However, there was no significant difference in the distance condition and no interaction between context and distance. There was an interaction between context and laterality [*F*_(2, 46)_ = 7.55, *p* < 0.01, η^2^ = 0.25]. Simple-effect tests showed that taxonomic relations elicited a larger P2 than the thematic relations in the left (*p* = 0.002), middle (*p* < 0.001), and right sites (*p* < 0.001), implying that the effect of context on P2 amplitude was greater in middle and right sites than left sites.

#### N400 (360–440 ms)

**Figure 4:**
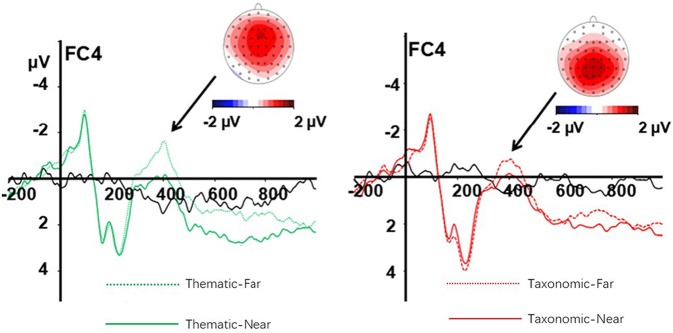
Difference waves and topographical maps of the distance effect for thematic relation **(left)** and taxonomic relation **(right)**. The green dot line is far thematic, the green solid line is near thematic, the red dot line is far taxonomic, the red solid line is near taxonomic, the black solid line is the difference wave.

Difference waves and topographical maps of the distance effect for thematic relation (left) and taxonomic relation (right) are shown in Figure 4. There was a main effect of context, *F*_(1, 23)_ = 5.02*, p* = 0.035, η^2^ = 0.18, and effect of distance, *F*_(1, 23)_ = 12.57, *p* = 0.002, η^2^ = 0.35. Thematic relations elicited a significantly larger N400 amplitude than taxonomic relations, and the far distance generally elicited a larger N400 amplitude than near distance.

An interaction between context and frontality was also observed [*F*_(4, 92)_ = 4.27, *p* = 0.003, η^2^ = 0.16]. A simple-effect analysis showed that thematic relations elicited a larger N400 amplitude than taxonomic relations in central (*p* = 0.014) and central-parietal (*p* = 0.015) areas, while the effect of context was non-significant in other sites (all *p*s > 0.05). In the same way, a three-way interaction of context, frontality, and laterality was observed [*F*_(8, 184)_ = 3.65, *p* = 0.001, η^2^ = 0.14]. A simple-effect analysis revealed an effect of context at the following sites: FC3, C3, Cz, CPz, Pz, C4, and CP4 (all, *p* < 0.05), with a larger N400 amplitude for thematic relations than for taxonomic relations.

There was a two-way interaction of distance and laterality [*F*_(2, 46)_ = 7.61, *p* = 0.001, η^2^ = 0.25]. A simple-effect analysis showed that far distance elicited a larger N400 amplitude than near distance conditions at the left (*p* = 0.005), middle (*p* = 0.001), and right sites (*p* = 0.002), implying that the effect of distance on N400 amplitude was greater in the middle and right sites than left sites. At the same time, there was a three-way interaction of context, distance, and frontality [*F*_(4, 92)_ = 8.37, *p* < 0.01, η^2^ = 0.27]. A simple-effect analysis showed a distance effect on thematic relation in the frontal (*p* = 0.013) and frontal-central (*p* = 0.011) regions, while the distance effect on taxonomic relations was found in the central-parietal (*p* = 0.001) and parietal (*p* = 0.001) regions.

#### LNC (500–800 ms)

To investigate the time course of the LNC more precisely, we analyzed three successive intervals, 500**–**600, 600**–**700, and 700**–**800 ms. Statistical analysis revealed a main effect of distance in all LNC latency windows (all *p* < 0.01). There was an interaction between distance and frontality in each latency window [*F*_500−600ms_ (4, 92) = 3.64, *p* = 0.008, η^2^ = 0.14; *F*_600−700ms_ (4, 92) = 2.83, *p* = 0.029, η^2^ = 0.11; *F*_700−800ms_ (4, 92) = 2.59, *p* = 0.042, η^2^ = 0.10]. Simple-effects tests showed that far distance evoked more negative waves than near distance conditions in all regions within each time window (500**-**600 ms: *p*_frontocentral_ = 0.031, *p*_central_ = 0.004, *p*_parietocentral_ = 0.002, and *p*_parietal_ = 0.003; 600**-**700 ms: *p*_frontocentral_ = 0.013, *p*_central_ = 0.003, *p*_parietocentral_ = 0.001, and *p*_parietal_ <0.001; 700**-**800 ms: *p*_frontocentral_ = 0.009, *p*_central_ = 0.001, *p*_parietocentral_ = 0.001, and *p*_parietal_ < 0.001), while the effect of distance on the LNC amplitudes was greater in posterior sites than in the anterior sites.

Within the 600**–**700 ms window, an interaction of distance and laterality was found [*F*_(2, 46)_ = 4.23, *p* = 0.021, η^2^ = 0.16]. A simple-effect analysis showed that far distance evoked greater negative waves than near distance conditions in the left (*p* = 0.018), middle (*p* = 0.002), and right sites (*p* = 0.003), implying that the effect of distance on LNC amplitude was greater in middle and right sites than left sites.

## Discussion

The main purpose of this study was to differentiate the electrophysiological response to inductive reasoning under thematic and taxonomic contexts. Behavioral results showed that there was a marginally significant difference of “yes” response between the two contexts when the distance is far; whereas the likelihood of a “yes” response was higher and the RT was shorter for the near distance compared to the far distance, which indicates that the reasoning process between the premise and conclusion required more effort in far distance than near distance conditions (Rips et al., 1973; Collins and Loftus, 1975; Green et al., 2010; Zawiszewski et al., 2011). The effect of distance was clearly found on the behavioral responses while the effect of context was only marginally significant when the distance is far. This is partially consistent with previous studies in that the performance for thematic relation is better than for taxonomic relation (Lin and Murphy, 2001; Sachs et al., 2008a; Vivas et al., 2016), particular when the object is artifacts (Kalénine et al., 2009; Lewis et al., 2015). By the merit of the high time resolution of ERP methods, we found that effects of distance and context were observed on the brain response to inductive arguments, which are discussed below.

ERP results revealed the effect of experimental condition in three time windows, corresponding to the P2, N400, and LNC components. In the P2 time window, there was an effect of context (thematic vs. taxonomic), but no effect of distance (near vs. far). During the N400 time window, both the context effect and distance effect were observed. In the LNC time window, only a distance effect was observed.

The difference between the two contexts of inductive reasoning were initially observed in the P2 time window. Taxonomic-based inductive reasoning elicited a larger P2 amplitude than thematic-based inductive reasoning trials. The P2 is generally associated with perceptual decoding (Li et al., 2009a; Liang et al., 2010; Wang et al., 2016) and early semantic processes (Lei et al., 2010; Maguire et al., 2010). In the present study, the P2 amplitude difference may reflect the different perceptual process (Collins and Loftus, 1975; Li et al., 2009a; Liang et al., 2010; Wang et al., 2016). Although we used linguistic stimuli in this study, participants may have implicitly activated the visual perceptual representations of the sematic meaning of words, possibly resulting in the increased processing of overlapping features in the low-level under the taxonomic condition compared with thematic condition. In addition, there are different levels of perceptual processing between thematic and taxonomic relationships. The formation of taxonomic relations is based on an overlap in perceptual features of category members (Kalénine et al., 2009). For instance, the close taxonomic relationship between saw and ax is that they are both made by metal and are both keen-edged. Therefore, it is necessary to compare common perceptual traits and other major behavioral characteristics between two objects or species to find the taxonomic relationship between them (Sachs et al., 2008a; Maguire et al., 2010; Schwartz et al., 2011; Chen et al., 2015a), which resulting in the larger P2 amplitudes. In contrast, when looking for the thematic relation between two species, participants have no need to compare the perceptual characteristics (Kalénine et al., 2009), but remember whether there is a thematic relationship between them.

It is necessary to note that there was only a context effect, but no distance effect on the P2 component. This indicates that, in an early time window such as that of the P2 component, participants distinguish relation types before proceeding to the next stage, semantic processing, which was associated with the N400. However, In the N400 time window, both a context effect (thematic vs. taxonomic) and distance effect (near vs. far) were observed. This indicates that, after decoding the related feature of two categories (premise and conclusion) within the P2 time window, participants made an elaborative semantic integration of the relation between premise object and conclusion object, and determined whether the conclusion object had the same property as the premise object. Interestingly, the P2 amplitude is larger for taxonomic than thematic relations while the opposite is true for the N400 amplitude. This implies that the thematic relation is not intensively processed in the earlier time window (e.g., P2) but is processed in the later time window, in which the knowledge-based relation is retrieved and integrated.

The N400 component is typically related to semantic integration (Long et al., 2015; Wamain et al., 2015) and semantic anomalies (Pijnacker et al., 2010). Incoherent words or sentences always evoke larger-amplitude N400 components (Gunter et al., 1995; Sachs et al., 2008a,b; Hickey et al., 2010; Chen et al., 2013). The N400 has also been observed in category-based inductive reasoning (Long et al., 2015; Wang et al., 2016). During reasoning, the concept is the basic unit, and humans principally conduct reasoning using conceptual information (Lau et al., 2008). Two concepts do not form a semantic relation until they have an intersection. Semantic distance is an influencing factor for inferring with the degree of relation between two concepts (Den Heyer and Briand, 1986). Kmiecik and Morrison (2013) investigated verbal analogical reasoning with different semantic distances, and found that near analogical distance elicits less negative N400 components than does far analogical reasoning (Kmiecik and Morrison, 2013).

In the present study, we used an inductive reasoning task and manipulated the context and distance between premise and conclusion. We found that, for both taxonomic and thematic conditions, far relation elicited larger N400 amplitudes than near relation reasoning. This result is consistent with existing studies on analogical reasoning, which suggests that semantic distance has a significant effect on reasoning within the N400 time window (Rips et al., 1973; Green et al., 2010). That is, in near distance conditions, it is easier to integrate and infer semantic relations between a premise and a conclusion. In contrast, for far distance, it is difficult to identify the intersection between two concept nodes, which evokes a larger N400 amplitude than that of near distance conditions.

Although distance effects were observed in the N400 for both two types of reasoning, the distance effect on thematic-based reasoning was mainly observed in the anterior (i.e., frontal and frontal-central) regions, while distance effect on taxonomic-based reasoning was observed in the posterior (i.e., central-parietal and parietal) region. Previous studies have shown that inductive reasoning mainly involves the left medial frontal or the left frontal gyrus (Goel and Dolan, 2004; Long et al., 2015) and an effect of semantic distance on analogical reasoning was found in the left frontopolar cortex (Goel and Dolan, 2004; Green et al., 2010; Long et al., 2015). Consistent with previous studies, the processing of thematic relationships is associated with activation of the prefrontal cortex (Hickey et al., 2010). However, taxonomic-based reasoning involved the process of comparing and analyzing critical or typical features of the objects in the premise and conclusion. The result of the present study supports the finding that the comparison of perceptual characteristics is primarily associated with activation of the parietal cortex (Bigman and Pratt, 2004).

In conclusion, a modified inductive reasoning task was used to investigate the electrophysiological difference between thematic and taxonomic-based inductive reasoning. ERP results revealed a significant effect of distance on both types of reasoning during the N400 time window, but the scalp distribution of this distance effect was different between these two types of semantic processing, with an anterior distribution for thematic-based reasoning and a posterior distribution for taxonomic-based reasoning. This supports the view that these two types of semantic processing may recruit different neural networks.

One possible confound for our results is that different sentences were used in the different conditions. People may be able to perceive the sentences in one of the conditions more easily and less easily in other condition. Though a large number of items were used in each condition, reducing the cause for concern. In future studies, the same sentence should be used as the “second sentence” across conditions to completely exclude this confound.

## Data Availability

The raw data supporting the conclusions of this manuscript will be made available by the authors, without undue reservation, to any qualified researcher.

## Ethics Statement

The experiment was approved by the ethics review board of Jiangxi Normal University.

## Author Contributions

FLi and LZ contributed to the conception and design of the study. FLiu organized the database and performed the statistical analysis and wrote the first draft of the manuscript. All authors contributed to manuscript revision, read, and approved the submitted version and approved the final version of the manuscript.

### Conflict of Interest Statement

The authors declare that the research was conducted in the absence of any commercial or financial relationships that could be construed as a potential conflict of interest.
