# Supplementary material for: Inductive Reasoning Differs Between Taxonomic and Thematic Contexts: Electrophysiological Evidence
Source: Front Psychol. 2019 Jul 25;10:1702. doi: 10.3389/fpsyg.2019.01702 (PMC6669940; doi:10.3389/fpsyg.2019.01702)
Supplement: Supplementary file 1 [file Table_1.docx]

***Appendix*** S*-Table 1. Linguistic stimuli used in the present study*

| **Premise** | | | **Conclusion** | | | | | | | | | | | |
| --- | --- | --- | --- | --- | --- | --- | --- | --- | --- | --- | --- | --- | --- | --- |
|  |  |  | **Taxonomic-near** | | | **Taxonomic-far** | | | **Thematic-near** | | | **Thematic-far** | | |
| Chinese | Pinyin | *English* | Chinese | Pinyin | *English* | Chinese | Pinyin | *English* | Chinese | Pinyin | *English* | Chinese | Pinyin | *English* |
| 锯子 | Ju zi | *Saw* | 斧头 | Fu tou | *Axe* | 钳子 | Qian zi | *Pliers* | 木头 | Mu tou | *Wood* | 钢筋 | Gang jin | *Rebar* |
| 茶叶 | Cha ye | *Tea leaf* | 咖啡 | Ka fei | *Coffee* | 沙冰 | Sha bing | *Smoothie* | 茶具 | Cha ju | *Teaware* | 瓷碗 | Ci wan | *bowl* |
| 奶茶 | Nai cha | *Milky tea* | 果汁 | Guo zhi | *Fruit juice* | 啤酒 | Pi jiu | *Beer* | 吸管 | Xi guan | *Sucker* | 黄油 | Huang you | *Butter* |
| 电影 | Dian ying | *Movie* | 动漫 | Dong man | *Animation* | 新闻 | Xin wen | *News* | 演员 | Yan yuan | *Actor* | 屏幕 | Pin mu | *Screen* |
| 项链 | Xiang lian | *Necklace* | 手镯 | Shou zhuo | *Bracelet* | 佛珠 | Fo zhu | *Bead* | 脖子 | Bo zi | *Neck* | 手腕 | Shou wan | *Wrist* |
| 扫帚 | Sao zhou | *Broom* | 拖把 | Tuo ba | *Mop* | 抹布 | Ma bu | *Duster* | 垃圾 | La ji | *Rubbish* | 灰尘 | Hui chen | *Dust* |
| 冰箱 | Bing xiang | *Refrigerator* | 空调 | Kong tiao | *Air conditioner* | 厨具 | Chu ju | *Kitchen* | 雪糕 | Xue gao | *Ice cream* | 猪肉 | Zhu rou | *Pork* |
| 喷嚏 | Pen ti | *Hiccup* | 打嗝 | Da ge | *Burp* | 懒腰 | Lan yao | *Lazy waist* | 鼻子 | Bi zi | *Nose* | 感冒 | Gan mao | *Cold* |
| 轮船 | Lun chuan | *Steamer* | 汽车 | Qi che | *Automobile* | 动车 | Dong che | *Bullet train* | 大海 | Da hai | *Ocean* | 孤岛 | Gu dao | *Island* |
| 牛排 | Niu pai | *Steak* | 鸡排 | Ji pai | *Chicken cutlet* | 火腿 | Huo tui | *Ham* | 餐叉 | Can cha | *Fork* | 牙签 | Ya qian | *Toothpick* |
| 国王 | Wang guo | *King* | 皇帝 | Huang di | *Emperor* | 奴隶 | Nu li | *Slave* | 城堡 | Cheng bao | *Castle* | 草屋 | Cao wu | *Cottage* |
| 镰刀 | Lian dao | *Sickle* | 锄头 | Chu tou | *Hoe* | 铁锹 | Tie qiu | *Spade* | 杂草 | Za cao | *Weeds* | 小麦 | Xiao mai | *Wheat* |
| 绷带 | Beng dai | *Bandage* | 纱布 | Sha bu | *Gauze* | 棉球 | Mian qiu | *Cotton ball* | 伤口 | Shang kou | *Wound* | 残疾 | Can ji | *Disability* |
| 警察 | Jing cha | *Police* | 军人 | Jun ren | *Soldier* | 教师 | Jiao shi | *Teacher* | 小偷 | Xiao tou | *Thief* | 证人 | Zhen ren | *Witness* |
| 蛋糕 | Dan gao | *Cake* | 面包 | Mian bao | *Bread* | 月饼 | Yue bing | *Moon cake* | 蜡烛 | La zhu | *Candle* | 雕花 | Diao hua | *Decorative cakes* |
| 火车 | Huo che | *Train* | 汽车 | Qi che | *Car* | 帆船 | Fan chuan | *Sailboat* | 铁轨 | Tie gui | *Railway* | 站台 | Zhan tai | *Platform* |
| 口红 | Kou hong | *Lipstick* | 唇膏 | Cun gao | *Lipstick* | 眼线 | Yan xian | *Eyeliner* | 嘴唇 | Zui chun | *Lip* | 额头 | E tou | *Forehead* |
| 婚纱 | Hun sha | *Wedding dress* | 西装 | Xi zhuang | *Suit* | 内裤 | Nei ku | *Briefs* | 新娘 | Xin niang | *Bride* | 新郎 | Xin lang | *Bridegroom* |
| 轻舟 | Qing zhou | *C*[*anoe*](javascript:;) | 帆船 | Fan chuan | *Sailboat* | 游艇 | You ting | *Yacht* | 湖水 | Hu shui | *Lake* | 船桨 | Chuan jiang | *Quant* |
| 婚宴 | Hun yan | *Wedding party* | 晚会 | Wang hui | *Party* | 庆典 | Qing dian | *Celebration* | 司仪 | Si yi | *Emcee* | 宾客 | Bin ke | *Guest* |
| 将军 | Jiang jun | *General* | 士兵 | Shi bing | *Soldier* | 文员 | Wen yuan | *Clerk* | 盔甲 | Kui jia | *Corselet* | 战马 | Zhan ma | *War-horse* |
| 台灯 | Tai deng | *Table lamp* | 书桌 | Shu zhuo | *Desk* | 床头 | Chuang tou | *Bedside* | 风扇 | Feng shan | *Air fan* | 音响 | Yin xiang | *Sound* |
| 钻戒 | Zhuan jie | *Diamond ring* | 手链 | Shou lian | *Bracelet* | 簪子 | Zan zi | *Hair clasp* | 中指 | Zhong zhi | *Midfinger* | 拇指 | Mu zhi | *Thumb* |
| 梳子 | Shu zi | *Comb* | 发夹 | Fa jia | *Hairpin* | 塑料 | Su liao | *Plastic* | 头发 | Tou fa | *Hair* | 指环 | Zhi huan | *Ring* |
| 丝袜 | Si wa | *Stockings* | 袜子 | Wa zi | *Socks* | 手套 | Shou tao | *Glove* | 双脚 | Shuang jiao | *Feet* | 头部 | Tou bu | *Head* |
| 面膜 | Mian mo | *Mask* | 湿巾 | Shi jin | *Wipes* | 香波 | Xiang bo | *Shampoo* | 脸颊 | Lian jia | *Cheek* | 手臂 | Shou bi | *Arm* |
| 钥匙 | Yao shi | *Key* | 门闩 | Men shuan | *Bolt* | 门铃 | Men ling | *Doorbell* | 铁锁 | Tie suo | *Caribiner* | 铁链 | Tie lian | *Shackles* |
| 瓦罐 | Wa guan | *Crock* | 铁锅 | Tie guo | *Iron pan* | 盘子 | Pan zi | *Plate* | 炖汤 | Dun tang | *Stew* | 火炉 | Huo lu | *Stove* |
| 裤子 | Ku zi | *Trousers* | 衬衫 | Cun shan | *Blouse* | 外套 | Wai tao | *Coat* | 皮带 | Pi dai | *Belt* | 草绳 | Cao sheng | *Straw rope* |
| 扁担 | Bian dan | *Shoulder pole* | 木棍 | Mu gun | *Club* | 铁棒 | Tie bang | *Peeler* | 水桶 | Mu tong | *Bucket* | 货物 | Huo wu | *Cargo* |
| 枕头 | Zhen tou | *Pillow* | 被子 | Bei zi | *Quilt* | 睡袋 | Shui dai | *Sleeping bag* | 脑袋 | Nao dai | *Head* | 棉絮 | Mian xu | *Cotton fiber* |
| 皮筋 | Pi jin | *Rubber hand* | 绳子 | Sheng zi | *String* | 电线 | Dian xian | *Electric wire* | 辫子 | Bian zi | *Pigtail* | 游戏 | You xi | *Game* |
